# Anticipation from sensation: using anticipating synchronization to stabilize a system with inherent sensory delay

**DOI:** 10.1098/rsos.171314

**Published:** 2018-03-14

**Authors:** Henry Eberle, Slawomir J. Nasuto, Yoshikatsu Hayashi

**Affiliations:** Brain Embodiment Laboratory, Biomedical Engineering, School of Biological Sciences, University of Reading, Reading RG6 6AH, UK

**Keywords:** anticipating synchronization, synchronization, time delay, robotic manipulator, state predictor

## Abstract

We present a novel way of using a dynamical model for predictive tracking control that can adapt to a wide range of delays without parameter update. This is achieved by incorporating the paradigm of anticipating synchronization (AS), where a ‘slave’ system predicts a ‘master’ via delayed self-feedback. By treating the delayed output of the plant as one half of a ‘sensory’ AS coupling, the plant and an internal dynamical model can be synchronized such that the plant consistently leads the target’s motion. We use two simulated robotic systems with differing arrangements of the plant and internal model (‘parallel’ and ‘serial’) to demonstrate that this form of control adapts to a wide range of delays without requiring the parameters of the controller to be changed.

## 1. Introduction

Closed-loop control is ubiquitous precisely because it is so useful—negative feedback allows a system to remain stable in the face of disturbance and continue functioning even in a changing environment. It is no surprise, then, that negative feedback loops are found so often in living organisms, which must cope with uncertain environmental conditions—the very concept of homeostasis is predicated upon them. However, some processes such as motor control are difficult to explain: closed-loop control is highly sensitive to feedback delay, and delays in the nervous system would not seem to support the quick movements that animals like humans make routinely. Some theorists argue that this can be reconciled through ‘strong anticipation’ [1], where a continuous coupling between the controlling process within the nervous system and the body itself allows otherwise delayed-feedback to be predicted. This also suggests a useful paradigm for the control of artificial systems as well, but practical examples of how a strongly anticipating system could be constructed are lacking. We have designed a framework for using the paradigm of anticipating synchronization (AS) to enable closed-loop control in the presence of uncertain or variable delays that we call the ‘sensory coupling’, which we believe displays the hallmarks of strong anticipation, and applied it to a classic control task of tracking a moving target with a simple robot arm.

**Figure 1.**
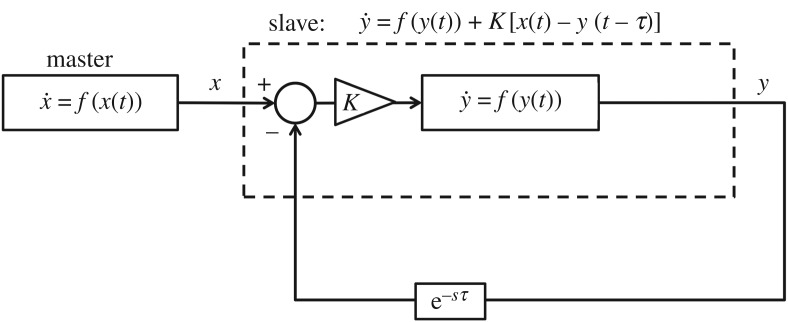
A generalized AS configuration based on delayed slave self-feedback, as described in [6]. The master (*x*) and slave (*y*) are governed by the same dynamics, save for the coupling term *K*[*x*(*t*)−*y*(*t*−*τ*)]. This term effectively increases the time constant of the slave, causing it to evolve more quickly than the master until *y*(*t*)=*x*(*t*+*τ*), at which point the coupling term reduces to 0 and the slave is synchronized with the future value of the master. If the master and slave dynamics are merely similar, not identical, synchronization can occur, but the coupling will never fully disappear.

AS is an extension of the phenomenon of synchronization in dynamical systems, where a ‘slave’ system can be made to synchronize with the future, rather than the instantaneous, state of an identical (or similar, in terms of their vector fields) ‘master’. The evidence for an AS-like phenomenon in human motor behaviour comes from delayed-feedback manual tracking experiments where human subjects attempted to synchronize the motion of their hands with a moving cursor. Stepp conducted one such study [2] in which subjects were instructed to track a chaotically oscillating target using a cursor that showed a delayed version of their movement. Subjects led (anticipated) the target in proportion to this delay until it reached a maximal value, rather than anticipating to the maximum degree immediately. This result is corroborated by multiple earlier studies that used similar methodology ([3–5]). Stepp found this consistent with a type of AS system identified by Voss [6], where anticipation is caused by a coupling term *K*[*x*(*t*)−*y*(*t*−*t*_*delay*_)] added to the slave, as in equation (1.2) and figure 1:

1.1 $$\dot{x}(t)=f(x(t))$$

and

1.2 $$\dot{y}(t)=f(y(t))+K[x(t)-y(t-\tau )],$$

but only if *τ* represents the feedback delay imposed by the experiment. A later collaboration between Stepp and Voss even indicated that so long as the continuous coupling with the body exists, the slave does not need to be similar to the master, and similar results can be achieved by a filter-like system that exhibits negative group delay [7].

This is in direct opposition to the theory postulating that motor control is dependent on a series of ‘forward models’ that fully replace the real, delayed sensory input with an estimate that is learned over time [8,9]. Miall & Wolpert [10] argued this explains how humans can perform fast movements that should be hindered by their physiological delays; the model is feed-forward, and does not need to wait for input from the body. This has not stopped AS potential for control theory applications from being recognized by some authors.

However, where AS has been applied to control it has been used within standard existing control frameworks rather than in an attempt to replicate strong anticipation. Oguchi & Nijmeijer [11] demonstrated that an AS slave system with a specified anticipation period could be inserted into a control loop, stabilizing it analogously to a Smith predictor [12,13]. The delayed-feedback is fully replaced by a predicted signal, allowing the controller to be designed as if the delay does not exist, but in principle the slave and its self-feedback could be replaced by a feed-forward model that estimates some *n* time steps ahead.

Although AS can be used within predictive control as shown by Oguchi and Nijmeijer, existing predictive controllers cannot be used to implement or replace a strongly anticipating AS controller. Firstly, there is no one element within the AS paradigm that can be replaced with a predictive model, as anticipation is a result of the continuous interaction between the master and slave. Secondly, and more importantly, there is no single predictive controller with fixed parameters that would exhibit equivalent behaviour to the strongly anticipating control proposed by Stepp and Voss, where the controller anticipates in proportion to the real feedback delay within the system.

We hypothesize that a control scheme based directly on the strong anticipation principle and using AS can be designed by coupling a correctly designed dynamical model to the real output of the plant through the sensors—a sensory coupling. Because the sensed output of the plant is subject to both, the delays introduced by the plant itself (feedback delay, *τ*_f_), and those that result from sensory processing (sensory delay, *τ*), the anticipation period will always be equal to the true delay within the system. The logic of this principle is similar to that of ‘signal bouncing’ [14], which avoids modelling network delay by ‘bouncing’ the output of a predictive model through the delaying channel itself.

We test our hypothesis by using a simulated robot (modelled in Simulink) to perform a tracking task in the presence of delay. Because of the challenges specific to this application, namely that the plant can only be controlled by a torque signal that is defined in a different coordinate system to the target, there are restrictions on how this coupling can be applied. Nonetheless, we believe we identified the two most plausible systems.

The ‘parallel’ system couples the plant itself to an internal model that encodes the ‘normal’ response of the control loop without delays. With both the model and plant tracking the same target, the plant synchronizes with the future state of the model, anticipating by a sufficient amount to counteract the delays in the real system.

The ‘serial’ system treats the moving target as the master, with an internal model predicting its motion. The control signal that corresponds to this predicted target is calculated and used to control the plant. The output of the plant, subject to the real system delays, forms the ‘slave’ part of the sensory coupling and ensures the degree of anticipation is always appropriate. In both of these cases, anticipation cannot occur without continuous interaction with the real target and plant, fulfilling on a basic level the requirements of strong anticipation.

The stability and tracking accuracy of the parallel and serial systems are tested and discussed in §§3 and 4, respectively. In addition to being compared with each other, comparisons are made with an unmodified control loop without anticipation 2 and one that does use AS, but without the new sensory coupling.

## 2. Common system elements

### 2.1. Common control loop dynamics

**Figure 2.**
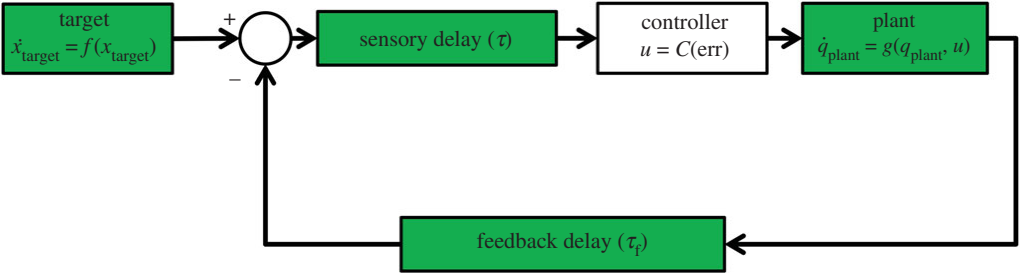
Block diagram of the basic control loop, without any form of anticipation applied. Green blocks represent unchangeable elements of the underlying system. The controller transforms the Cartesian difference between the position of the target and the end-effector of the robotic arm plant into appropriate motor torques using equation (2.7). The sensory delay term (*τ*) represents a delay in processing all sensory information, while the feedback delay term (*τ*_f_) represents a delayed reaction from the plant.

We present modifications to an underlying closed-loop control system, seen in figure 2. The plant is a simple two-link planar arm, modelled as two thin rods connected by frictionless revolute joints:

2.1 $$\ddot{q}=R{\left(q\right)}^{-1}(\tau -S(q,\dot{q})\dot{q}),$$

where ***q***, $\dot{q}$ and $\ddot{q}$ are the rotational joint position, velocity and acceleration, respectively, ***τ*** is the torque at the joints, ***R***(***q***) is an inertia matrix and $S(q,\dot{q})$ is a vector of Coriolis forces.

The Cartesian position of the arm’s end-effector (***x***_effector_) is related to the rotational position of its joints (***q***) by the forward kinematic equation:

2.2 $$x_{\mathrm{effector}1}=l_{1}\cos\left(q_{1}\right)+l_{2}\cos\left(q_{1}+q_{2}\right)$$

and

2.3 $$x_{\mathrm{effector}2}=l_{1}\sin\left(q_{1}\right)+l_{2}\sin\left(q_{1}+q_{2}\right),$$

where *l*_1_ and *l*_2_ are the lengths of the rods that compose the arm.

The target’s motion follows the *x* and *y* terms of a chaotic Rössler system, governed by equations (2.4)–(2.6), such that *x*_target1_=*x* and *x*_target2_=*y*:

2.4 $$\begin{array}{rl} \dot{x} & \;=-y-z, \end{array}$$

2.5 $$\begin{array}{rl} \dot{y} & \;=x-0.15y \end{array}$$

2.6 $$\begin{array}{rl} \text{and}\;\dot{z} & \;=0.2+z(x-10). \end{array}$$

**Figure 3.**
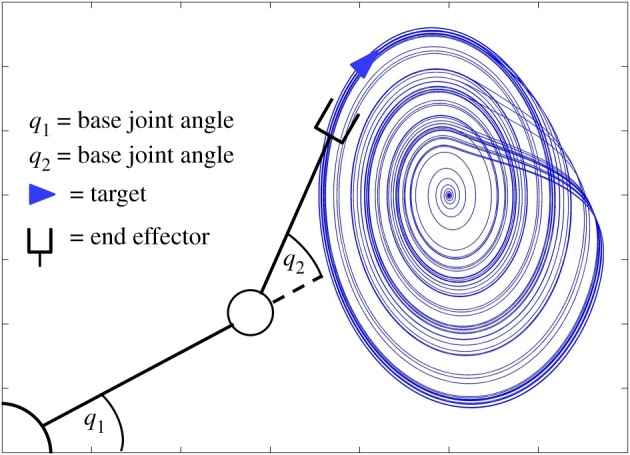
Illustration of the task performed by the simulated robot arm: the target, represented by a blue arrow, moves along a chaotic trajectory, while the controller changes the joint angles *q*_1_ and *q*_2_ of the arm in order to track it with the end-effector. The length of each link is set at 4, while their masses are set at 5 (in arbitrary units).

The arm must track the target, meaning that the end-effector of the arm must maintain the target’s position and velocity (figure 3). This is achieved through the use of a simple proportional-derivative control law based on the Jacobian transpose method, which is a well-documented means of transforming a Cartesian error into appropriate torques at the joints [15]:

2.7 $$\tau =J^{T}(K_{p}(x_{\mathrm{target}}-x_{\mathrm{effector}})-K_{v}{\dot{x}}_{\mathrm{effector}}),$$

where ***K***_p_ and ***K***_v_ are the proportional and derivative gain terms, and ***J*** is the derivative of the arm’s forward kinematics (equation (2.3)) with respect to the joint angles ***q***.

### 2.2. Simulation parameters

We simulate the control loop using Matlab Simulink with a ode3 Bogacki-Shampine fixed-step solver at a time resolution of 0.001 s. The control loop performance is hindered by two distinct ‘problematic’ delays, both modelled by a Simulink variable-time transport delay block. Firstly, a delay (*τ*) is applied to output from both the target and plant, representing an unavoidable sensory processing delay. The feedback delay (*τ*_f_) applies only to the feedback from the plant, and represents an additional delay between the plant receiving the control signal *u* and its observable response. These both represent delays in the controlled system that ordinarily prevent stability and must be overcome through the introduction of the AS paradigm in the form of the sensory coupling.

### 2.3. Predictive tracking problem

The aim for the two sensory coupling-based models is to effectively compensate for *τ* and *τ*_f_ through anticipation, ensuring that the robot neither lags the target nor loses tracking accuracy when subject to these delays. As such, their performance is compared against that of the original control loop (figure 2) when *τ* and *τ*_f_ are both 0. Lag (or lead, when successfully anticipating the target) is measured in seconds and calculated by cross-correlating the *x*-axis positions of the target and end-effector over 200 s of simulated tracking and finding the time lag (or lead) of the maximum correlation coefficient. The tracking performance is given by the root mean square error (RMSE) between target and end-effector at the previously discovered lag/lead (lag-adjusted RMSE):

$$E_{\mathrm{RMS}}=\left\{ \begin{array}{ll} \sqrt{\frac{\sum_{t=|l|}^{T}(x_{\mathrm{target}}(t)-x_{\mathrm{effector}}(t-|l|))}{T-2|l|}} & l\geq 0,\\ \sqrt{\frac{\sum_{t=1}^{T-|l|}(x_{\mathrm{target}}(t-|l|)-x_{\mathrm{effector}}(t))}{T-2|l|}} & l<0, \end{array}\right.$$

where *T* is the length of the simulation and *l* is the lag term.

This makes it possible to distinguish behaviour that is merely anticipatory with behaviour that is anticipatory and accurate, which is our stated goal.

Without delays, and with the gain values ***K***_p_=70 and ***K***_v_=100, the lag-adjusted RMSE between the positions of the end-effector and target is 0.1024 (for comparison, the target’s distance from the origin varies between 0 and 2) and the lag is 0.01 s. The basic control loop becomes increasingly unstable as delay is increased, with unstable oscillations masking any tracking behaviour at or above 0.045 s feedback or sensory delay.

## 3. Anticipation using plant dynamics (parallel system)

**Figure 4.**
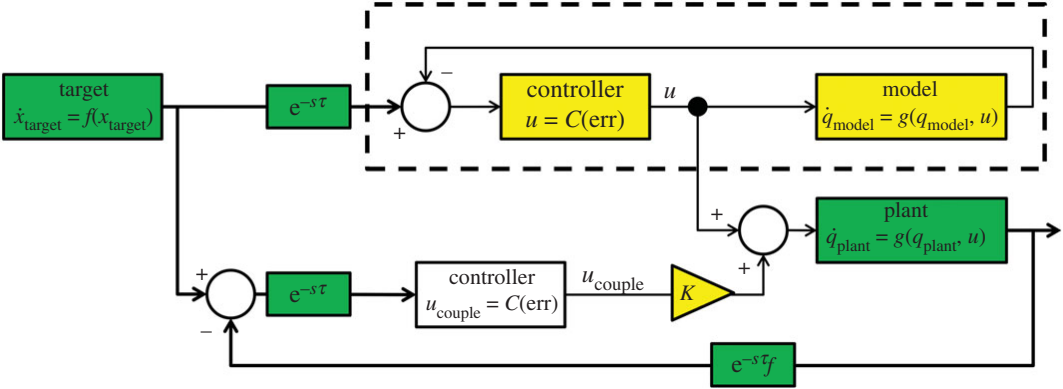
The parallel system. The dynamics of the plant (*g*(***q***_plant_,***u***)) and controller (***u***=*C*(err)) are replicated in an internal model (delineated by a dashed box) that uses instantaneous feedback to produce the primary control signal ***u***. Green blocks represent unchangeable elements of the underlying system, while yellow blocks have been added to enable anticipation. The delayed-feedback from the plant provides the ‘slave’ component for the coupling term, which is transformed by the controller into a secondary control signal (*u*_couple_) that is scaled by the coupling constant *K*. This drives the plant itself to anticipate the target.

In order to anticipate the target while using only the plant dynamics the plant and an internal model with identical dynamics are coupled in what we call a ‘parallel’ configuration, which can be seen in figure 4. This is based on the principle that anticipation can occur between non-autonomous dynamics that share a common driver [16,17]. In the parallel system, the plant anticipates the internal model, which is itself driven to follow the target—the result is that the plant anticipates the target by a small amount.

As stated in the introduction, information from the plant is affected by an associated sensory delay, *τ* and feedback delay, *τ*_f_. However, the internal model is not, and because of its instantaneous feedback it is used to calculate the control signal for both itself and the plant, and is treated as the AS master. The plant’s state acts as the delayed self-feedback necessary for the AS coupling, designating it as the slave. Because the plant is a robot that must be controlled by applying torque at its joints, the control law in equation (2.7) (with gains of ***K***_p_=70 and ***K***_v_=100) transforms the difference between the target and plant positions into an appropriate coupling torque that is added to the plant’s control input. This coupling torque drives anticipation in the plant and compensates for discrepancies between the control signal based on the internal model and the actual state of the plant.

We expect the sensory coupling to increase the system’s robustness to disturbance in the plant, as opposed to a controller based only on an internal model, so the relationship between coupling strength and stability is examined in addition to anticipation.

### 3.1. Testing

In order to find the limits of the parallel system’s anticipation, the tracking task was simulated over a range of values for *K* and both *τ* and *τ*_f_, and the lag and lag-adjusted RMSE. This allows the region in which stable anticipation (and thus, accurate tracking) occurs to be plotted as a function of coupling strength and delay. The system was also exposed to a sensory delay that was abruptly doubled in length partway through a movement in order to test its robustness to non-constant delay.

Finally, the system was again simulated over a range of *K* and *τ* values, this time perturbing the plant with a large torque impulse and recording the RMSE immediately afterwards in order to determine the coupling’s effect on stability.

### 3.2. Results

**Figure 5.**
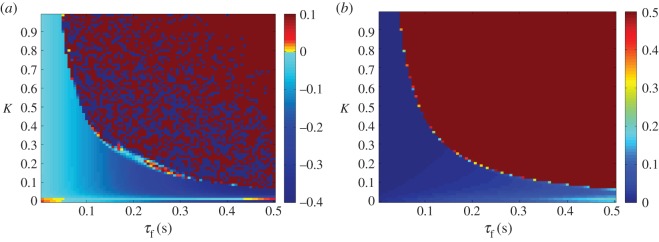
Lag (*a*) and lag-adjusted RMS error (*b*) between *x*_target_ and *x*_effector_, plotted as a heat map. *τ* is set to 0 s, while *τ*_f_ is varied between 0.01 and 0.5 s. Anticipation (seen here as blue, negative lag values) increases with *τ*_f_, reaching its maximum near the stability boundary (which can be approximated by *Kτ*_f_=0.14) beyond which error rises above acceptable levels and lag can no longer be accurately measured. For this reason, the error is saturated at 0.5.

**Figure 6.**
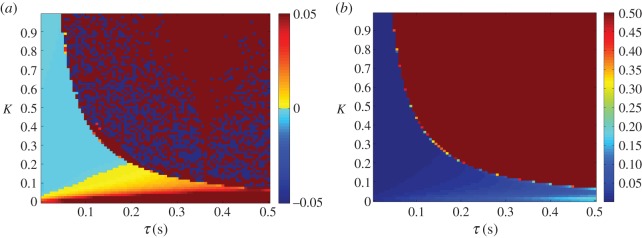
Lag (*a*) and lag-adjusted RMS error (*b*) between *x*_target_ and *x*_effector_, plotted as a heat map. *τ*_f_ is set to 0 s, while *τ* is varied between 0.01 and 0.5 s. Lag remains close to 0 to the left of the stability boundary (which can be approximated by *Kτ*=0.14), beyond which error rises above acceptable levels and lag can no longer be accurately measured. For this reason, the error is saturated at 0.5.

**Figure 7.**
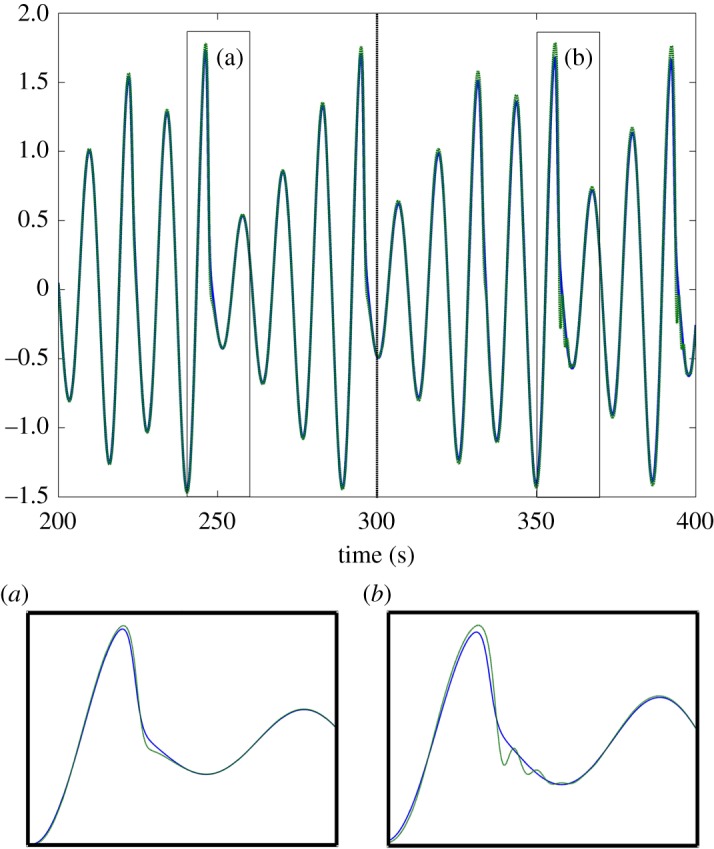
*x*-axis movement of the target (blue, solid line) and the end-effector of the parallel system plant with *K*=0.3 (green, dashed line), which must track it. *τ*=0.1 *s* before rising to 0.3 s at the 300 s point. The increase in *τ* causes the end-effector to oscillate more after non-sinusoidal moments of the target (cf. inset figures (*a*) and (*b*)), but does not cause a proportionate increase in lag.

As the *τ*_f_ is increased in this system, the plant begins leading the target as hypothesized (this can be seen in figure 5*a*). This is associated with a decrease in tracking accuracy however; once the feedback delay has reached 0.5 s the error has grown noticeably (figure 5*b*). When *τ* is increased, as seen in figure 6*a*, the lag changes very little over the stable area, which is the same as in the previous condition. This reflects that the plant’s anticipation is near equal to the delay on the target information. This holds even if the delay increases during the movement. Figure 7 shows how a mid-execution doubling of sensory delay does not affect the system’s lag, although the increase in tracking error can be observed in specific peaks of the end-effector’s motion (insets a and b).

**Figure 8.**
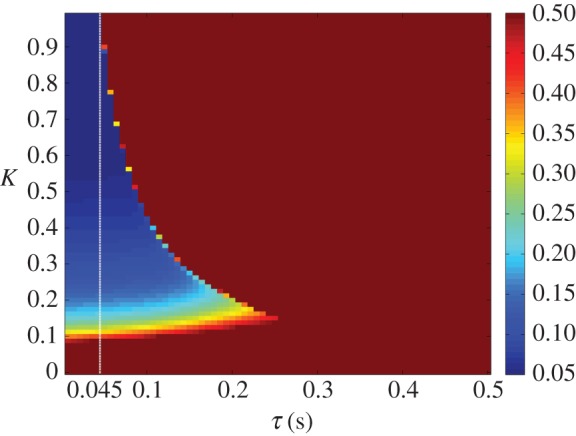
RMSE in the 100 s after an impulse torque disturbance of magnitude 700 is applied at both joints, plotted as a function of *K* and *τ* on a heat map. The heat map is saturated above an error of 0.5, representing a failure to track the target. Higher values of *K* reduce the effect of the disturbance, meaning that the stable region is bounded by the approximate *Kτ*=0.14 stability boundary, as well as a lower limit in *K* of approximately 0.1. A white dotted line indicates the highest delay value (0.045 s) at which the original control loop is stable (subject to the same conditions).

**Figure 9.**
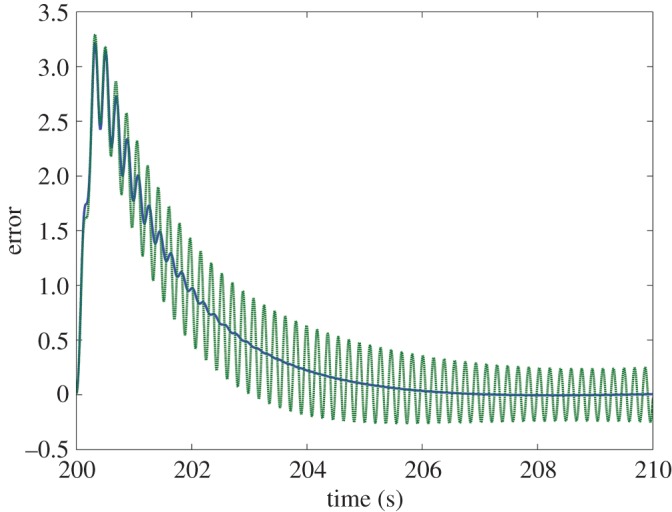
Instantaneous error between the positions of the target and the end-effector (*x*_target_−*x*_effector_) for the parallel system with *K*=0.9 (blue line) and the unmodified control loop (green dashed line) along the *x*-axis in response to a large impulse torque of magnitude 4000 and duration 0.3 s at both arm joints beginning at 200 s. Both systems are subject to the delay terms *τ*=0s and *τ*_f_=0.045 *s*. The parallel system exhibits a significantly reduced response, returning to stability within 5 s.

Finally, the coupling has a beneficial effect on the system’s ability to resist perturbation. As seen in figure 8, an increased *K* value makes the system more robust to a large impulse disturbance, even at low delays, and more so than the original control loop. This is clearly displayed in figure 9, where it can be seen that the response of the parallel system to disturbance is much smaller in magnitude.

### 3.3. Discussion of parallel system performance

In the parallel system, the plant itself is turned into a predictor by treating its coupling with the internal model as an additional control signal. Through this sensory coupling, the internal model becomes the master and the plant the slave, a reversal of the relationship seen in the work of Oguchi & Nijmeijer [11] and indeed the general expectation that a predictor would be a separable element from the system under control. This is reminiscent of the principle of morphological control [18]: some elements of the predictive control task have been transferred from the controller into the controlled object and anticipation cannot occur without both.

The parallel system is very stable both to feedback delay and disturbance at the plant, becoming more so as the coupling strength is increased. Where the delay is 0, this is nothing more than an increase in control gain, but the mechanism becomes distinct as the delay increases. Correcting a disturbance can be thought of as a form of initial-value problem. A sufficiently strong coupling allows a slave system to synchronize with the master, even if their current states differ, which is the situation created by a disturbance to the plant.

Despite being more stable than the unmodified control loop (figure 2), the parallel system’s tracking accuracy declines as the delay increases, as seen in figure 6*b*, with a specific example visible in figure 7. In practice, since the slave is not a Rössler oscillator itself it cannot anticipate every element of the target’s motion, and particularly exhibits errors when this motion is non-sinusoidal. The reduced effectiveness of non-identical slaves is an identified limitation of AS and the effect can be limited by a closer match between the target and plant dynamics.

## 4. Anticipation using environmental dynamics (serial system)

**Figure 10.**
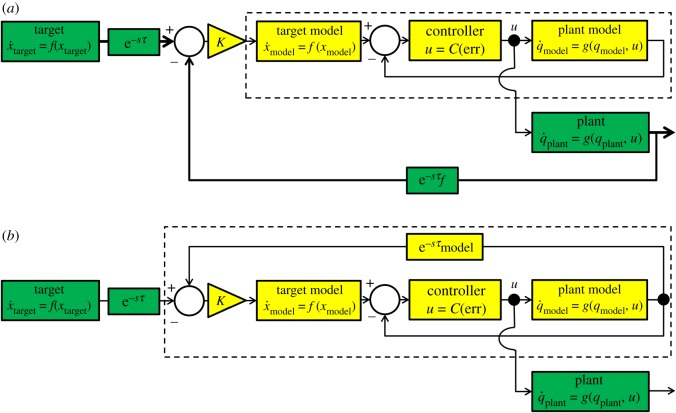
The serial system and an alternative system based on direct slave feedback. The internal slave model is delineated by a dashed box. Green blocks represent unchangeable elements of the underlying control loop, while yellow blocks have been added to enable anticipation. (*a*) The serial system. An internal model of the system’s dynamics acts as the ’slave’ in AS, while the output of the plant provides delayed self-feedback. The output of the model is a prediction of the future value of the err term, which is used to drive the controller without lag. (*b*) Alternative system where the delayed self-feedback is taken directly from the slave with an independent delay *τ*_model_ that must be tuned to closely match the sum of delays in the system. This entails the removal of feedback from the real slave.

In our second, ‘serial’, system the internal slave contains both the dynamics of the target and the plant, in order to anticipate the visual error term err (the difference between the positions of the target and end-effector). This term is fed directly into the controller, replacing the true delayed value of err, as seen in the block diagram shown in figure 10*a*.

The target is treated as the master, while the slave has the same dynamics as the system described in §2.1 and shown in figure 2, minus the sensory and feedback delays. This constitutes an autonomous system that can be synchronized with the target’s motion. The coupling term (equation (4.1)) is added directly to the $\dot{x}$ and $\dot{y}$ terms of the target dynamics within the slave (equation (2.4), equation (2.5)). The plant itself is treated as a delay between sensing the movement of the target and sensing the end-effector’s response. Theoretically, any sensory or actuation delays (within certain stability bounds) will be compensated by an equal period of anticipation:

4.1 $$K[x_{\mathrm{target}}(t-\tau )-x_{\mathrm{plant}}(t-\tau -\tau_{f})].$$

Since the target dynamics are incorporated in the slave, the serial system should be able to more accurately anticipate all features of the target’s movement and thus achieve better tracking performance than the parallel system.

We compare this with an approach where the state of the slave is delayed within the slave model (*τ*_model_) and fed directly fed back in the coupling, as in figure 10*b*, in order to demonstrate that our system is not vulnerable to applying anticipation that does not match the true system delay.

### 4.1. Testing

In order to find the limits of the serial system’s anticipation, the tracking task was simulated over a range of values for *K* and both *τ* and *τ*_f_, and the lag and lag-adjusted RMSE. This allows the region in which stable anticipation (and thus, accurate tracking) occurs to be plotted as a function of coupling strength and delay. The system was also exposed to a sensory delay that was abruptly doubled in length partway through a movement, and its response compared with that of the system described in figure 10*b*.

The system was simulated over a range of *K* and *τ* values while subject to a torque disturbance at a fixed point in each movement. The system stability was also compared against that of the original control loop by exposing both to a step disturbance and recording their response.

### 4.2. Results

**Figure 11.**
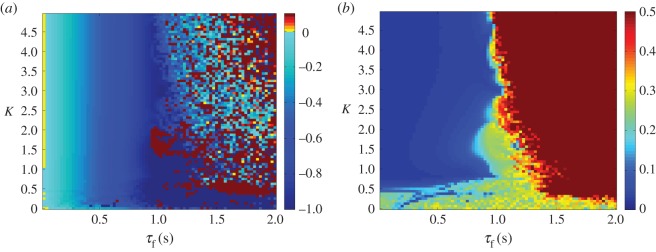
Lag (*a*) and lag-adjusted RMSE (*b*) between *x*_target_ and *x*_effector_, plotted as a heat map. *τ* is set to 0 s, while *τ*_f_ is varied between 0.01 and 2 s. The anticipation (seen as negative lag) closely matches *τ*_f_ within a near-rectangular region bounded by *K*>1 and *τ*_f_<1 *s*, while at higher delays tracking no longer occurs and lag cannot be accurately measured. For this reason, the error is saturated at 0.5.

The relationship between anticipation time, coupling strength and feedback delay can be seen in figure 11*a*. The lag between target and end-effector decreases and is replaced by a significant degree of anticipation as the feedback delay *τ*_f_ is increased. Unlike the parallel system, the tracking performance barely decreases within the region of stable anticipation, as seen in figure 11*b*.

**Figure 12.**
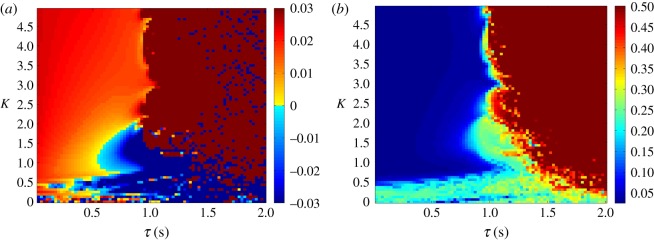
Lag (*a*) and lag-adjusted RMSE (*b*) between *x*_target_ and *x*_effector_, plotted as a heat map. *τ*_f_ is set to 0 s, while *τ* is varied between 0.01 and 2 s. The lag remains close to 0 s within a near-rectangular region bounded by *K*>1 and *τ*_f_<1 *s*, while at higher delays tracking no longer occurs and lag cannot be accurately measured. For this reason, the error is saturated at 0.5.

Where the feedback delay is set to 0 s, the lag remains close to 0 for a range of values of *τ*, as can be seen in figure 12*a*, indicating that the anticipation closely matches the delay. As shown in figure 12*b*, the dependence of the tracking accuracy on *τ* is the same as its dependence on *τ*_f_.

**Figure 13.**
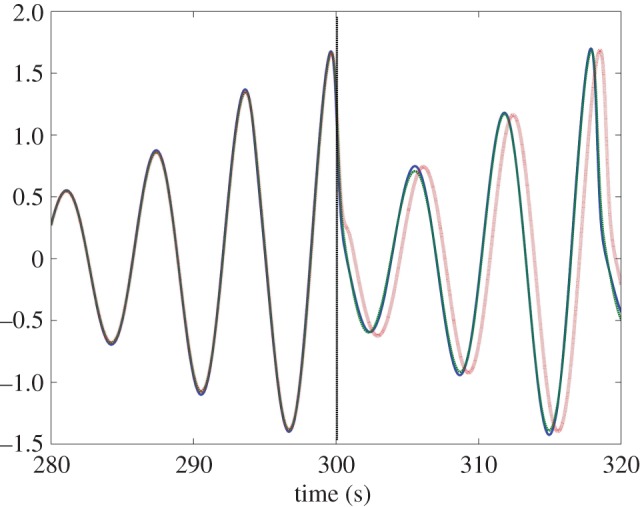
*x*-axis position of the target (blue, solid line), the end-effector of the serial system (green, dashed line) and the end-effector of the alternative system using direct slave feedback with *τ*_model_=0.3 (red, dotted line). In both cases, *τ*=0.1 *s* before rising to 0.7 s at the 300 s point (black, dashed vertical line) and *K*=1. To begin with, all three coincide in time, but after the increase in *τ* the system with direct slave feedback clearly lags (by the difference between *τ* and *τ*_model_).

The serial system is very robust to changes in delay. Even if *τ* doubles mid-movement, the behaviour of the system does not change, while a system based on internal delay falls out of step with the target (figure 13).

The serial system was found to be much less robust to disturbance than the parallel system when subject to the same tests, with even a small disturbance to the plant’s torque causing major instability.

### 4.3. Discussion of serial system performance

The serial system achieves a long period of highly accurate anticipation by replicating the full dynamics of the target system in the internal slave, meaning that even a chaotic target can be tracked with very high accuracy (as seen in figure 13). Because the true plant is assumed to behave identically to the matching dynamics within the slave, its output is treated as simply a delayed version of the slave’s state. Any disturbance to the plant will violate this assumption, and in practice, the serial system becomes unstable when subject to much smaller disturbances than the parallel, to the point that a counterpart to figure 9 could not be made.

This limitation is linked to the sensitivity to initial conditions of the chaotic dynamics that represent the target in the slave. These diverge exponentially from the target’s actual movement when the plant, and thus the sensory coupling, is disturbed. It would be possible to achieve anticipation using less-sensitive dynamics to represent the target within the slave, but these would no longer match the target dynamics and thus limit both the anticipation period and accuracy that can be achieved, resembling more the performance of the parallel system.

## 5. General discussion

In this work, we sought to find a ‘middle ground’ between two very different approaches to reconciling AS with the problem of controlling delayed systems. Oguchi and co-workers [11,14] adapted well-understood predictive control frameworks such that a feed-forward predictive model could be replaced by an AS slave with delayed self-feedback. By contrast, the works of Voss and Stepp identified a strong correspondence between human subjects’ ability to anticipate a moving target and the feedback delay they experienced and fitted a plausible high-level AS [2] (and later, anticipatory negative group delay [7]) model to this behaviour. With the parallel and serial systems based on the sensory coupling we aimed to apply the beneficial properties of the anticipatory systems designed in the latter approach (the ability to respond to a wide range of delays with the appropriate degree of anticipation) to a well-defined nonlinear control task—tracking a moving target with a planar two-joint robot arm.

Both the parallel and serial systems exhibited hallmarks of strong anticipation: no anticipation occurred unless there were delays present in the underlying control loop, and where it did occur it was proportional to that delay, as seen in figures 5, 6, 11 and 12. This is qualitatively the same behaviour that led Stepp to suggest AS as a principle employed in human motor control, and it is also uniquely useful for the control of artificial systems (such as robots), representing control that is both adaptable and robust. Continuous feedback from the plant allows compensation for multiple levels of delay without changing the gain of the controller or the parameters of the internal dynamical model (a concept outlined by the comparison in figure 13 between the serial system and its counterpart without this feedback).

In performing a tracking task an additional criterion was imposed, that in addition to exhibiting stable anticipation the robot’s motion must closely match that of the target. In this latter respect the serial system was more successful, showing low overall error over the majority of the parameter region in which it was stable, unlike the steady increase of error with delay shown by the parallel system (cf. figure 6*b* with figure 12*b*). This is unsurprising in that the serial system anticipates the target using a slave that contains identical dynamics, which previous studies on AS have suggested is a ‘gold standard’ for accurate prediction. However, in this case, the chaotic Rössler dynamics of the target were highly sensitive to external disturbance, which was inevitably transmitted through the sensory coupling, causing instability. This suggests that for control tasks, where robustness to disturbance is at least as important as robustness to delay, there may be trade-offs for fully accurate prediction.

The parallel system exhibited much greater robustness to disturbance and a lesser accuracy than the serial system, as can be seen in figure 8. This suggests that while the near-perfect prediction of the serial system would be appropriate for tasks where unexpected events will not occur, it is the parallel system that is more suited for robots performing ‘human-like’ tasks in unstructured environments, particularly because it is not limited to anticipating a single type of target. As many solutions exist for actuation in predictable environments, it would seem that the parallel system has a larger niche in the existing robotic control landscape.

## 6. Conclusion

We have introduced two closed-loop control systems that represent early applications of the principles of strong anticipation to robotic control, using the paradigm of AS. Thanks to a ‘sensory coupling’ between an internal model and the true dynamics of the plant, delays within the control loop are seamlessly counteracted by a matching degree of anticipation, ensuring that the robot’s response neither lags nor becomes unstable. This holds true even if the delay changes mid-execution, with no need to change a corresponding parameter within the internal model. In the ‘parallel’ system, the sensory coupling is fed to the plant concurrently (or ‘in parallel’) with the primary control signal to enable anticipation, while in the ‘serial’ system the same coupling drives the internal model to produce a primary control signal that anticipates the target.

Both of these proposed control schemes would be applicable to controlling a teleoperated robot, or one with slow actuation (such as a soft robot). In particular, the ‘parallel’ system exhibits robustness to both unpredictable delay and unmodelled disturbances, while its internal model’s dynamics are not specific to the chaotic target in this tracking task, meaning it is not limited to tracking only one possible target. This fact, combined with the fact that the serial system’s target-specific dynamics proved unstable in response to disturbances, indicates that strong anticipation through coupling with the body dynamics alone could be a useful paradigm for tasks in highly unstructured environments.

In addition to testing this new control methodology on a more complex robot model, this work could be greatly expanded by exploring the behaviour of these systems where their internal model does not match the dynamics of the plant or the target. Although counterintuitive, there is evidence that this could have a beneficial effect on the stability of the control, and would allow for a more generally applicable controller.

## Data accessibility

The Simulink models and Matlab code used to generate the results seen in this manuscript are archived at [19].
